# A validation method for near-infrared spectroscopy based tissue oximeters for cerebral and somatic tissue oxygen saturation measurements

**DOI:** 10.1007/s10877-017-0015-1

**Published:** 2017-04-03

**Authors:** Paul B. Benni, David MacLeod, Keita Ikeda, Hung-Mo Lin

**Affiliations:** 1 CAS Medical Systems (CASMED), Inc., Branford, CT USA; 2 Human Pharmacology & Physiology Lab, Department of Anesthesiology, Duke University Medical Center, Durham, NC USA; 3 Department of Anesthesiology, University of Virginia, Charlottesville, VA USA; 4 Department of Population Health Science and Policy, Icahn School of Medicine at Mount Sinai, New York, NY USA

**Keywords:** Tissue oximetry, Cerebral oximetry, Near Infrared Spectroscopy, NIRS, Tissue oxygen, Saturation, FDA

## Abstract

We describe the validation methodology for the NIRS based FORE-SIGHT ELITE^®^ (CAS Medical Systems, Inc., Branford, CT, USA) tissue oximeter for cerebral and somatic tissue oxygen saturation (StO_2_) measurements for adult subjects submitted to the United States Food and Drug Administration (FDA) to obtain clearance for clinical use. This validation methodology evolved from a history of NIRS validations in the literature and FDA recommended use of Deming regression and bootstrapping statistical validation methods. For cerebral validation, forehead cerebral StO_2_ measurements were compared to a weighted 70:30 reference (REF CX_B_) of co-oximeter internal jugular venous and arterial blood saturation of healthy adult subjects during a controlled hypoxia sequence, with a sensor placed on the forehead. For somatic validation, somatic StO_2_ measurements were compared to a weighted 70:30 reference (REF CX_S_) of co-oximetry central venous and arterial saturation values following a similar protocol, with sensors place on the flank, quadriceps muscle, and calf muscle. With informed consent, 25 subjects successfully completed the cerebral validation study. The bias and precision (1 SD) of cerebral StO_2_ compared to REF CX_B_ was −0.14 ± 3.07%. With informed consent, 24 subjects successfully completed the somatic validation study. The bias and precision of somatic StO_2_ compared to REF CX_S_ was 0.04 ± 4.22% from the average of flank, quadriceps, and calf StO_2_ measurements to best represent the global whole body REF CX_S_. The NIRS validation methods presented potentially provide a reliable means to test NIRS monitors and qualify them for clinical use.

## Introduction

The history of validations for tissue oxygen saturation (StO_2_) measurements of the brain dates back to 1991. McCormick et al. [1], first described the comparison of a Near-Infrared Spectroscopy **(**NIRS) monitor (INVOS^®^ 2910, Somanetics Corp. acquired by Medtronic, Dublin, Ireland) to a mixed bed of arterial, venous, and capillary blood in the brain, using a weighted blood reference consisting of both arterial and venous blood. Pollard et al. [2] validated the first US FDA cleared commercial NIRS cerebral oximeter (INVOS^®^ 3100, Somanetics Inc., acquired by Medtronic, Dublin, Ireland) with a weighted blood co-oximetry reference of 0.75 × jugular bulb oxygen saturation (SjbO_2_) and 0.25 × arterial oxygen saturation (SaO_2_) [3, 4]. Henson et al. [5] and Shah et al. [6] followed with similar comparison studies with the INVOS 3100 monitor. Several years later, the first-generation FORE-SIGHT cerebral oximeter was validated against a cerebral blood weighted reference of 0.70 × SjbO_2_ and 0.30 × SaO_2_, [7–10], which was supported by PET studies by Ito, et al. [11] Other NIRS cerebral oximeter validations on both adult and pediatric subjects adopted the weighted 70:30 SjbO_2_:SaO_2_ reference [12–14].

For tissue oxygen saturation (StO_2_) measurements of somatic (non-cerebral) locations, the reference used for NIRS monitor StO_2_ comparative and validation studies has been more varied and has included in-vitro comparisons. Research NIRS devices monitoring skeletal muscle were compared to a local venous oxygen saturation value from a blood draw during exercise [15–17]. The Hutchinson InSpectra™ (Hutchinson Technology Inc., Hutchinson, MN USA) was validated by comparing sensor measurements to blood saturation values in an in-vitro setup [18]. The ViOptix ODISsey™ (ViOptix, Inc, Fremont, CA USA) and Invos 3100 monitors were compared to co-oximetry measurements of blood draws on isolated animal limbs [19, 20]. For pediatrics, NIRS human somatic measurements were compared to central venous blood saturation values [21, 22]. Later, FORE-SIGHT pediatric somatic StO_2_ values were validated by comparing to a weighted blood co-oximetry weighted blood co-oximetry reference of 0.70 × central venous oxygen saturation (ScvO_2_) and 0.30 × arterial oxygen saturation (SaO_2_) [23], which was supported by Pang et al. [24] from estimating whole body venous volume ratio.

The purpose of this paper is to describe one methodology of validating NIRS based tissue oximeters accepted by the US FDA for adult clinical clearance. For other world regulatory bodies such as the European Union Medical Device Directive (93/42/EEC) [25], there are similar requirements for clinical clearance of medical devices. This methodology of validating NIRS based tissue oximeters was used to obtain clinical clearance in the European Union, Canada, Australia, China, Japan, and Russia. Although industry methods of validation and FDA requirements have generally converged in the last two decades, there is no universally accepted reference to compare tissue oximeters against. The US FDA currently prefers oximeter validations, whether pulse oximeters, or tissue oximeters to be compared to a blood reference. The US FDA 510(K) medical device clearance method requires a reference to one or more similar function predicate devices that are validated similarly to the new medical device being evaluated. We present the methodology behind the validation of the second-generation FORE-SIGHT^®^ tissue oximeter (FORE-SIGHT ELITE^®^, CAS Medical Systems, Branford, CT USA) for both cerebral and somatic tissue oxygen saturation (StO_2_) monitoring, with rationale behind the assumptions made, selection of a comparative reference, statistical methods used, subject recruitment requirements, particularly in terms of diverse skin tones, and regulatory requirements for clinical use. This NIRS validation methodology evolved from a history of NIRS-based tissue oximeter validation publications and FDA correspondence recommending use of Deming regression and bootstrap resampling techniques for analysis of comparative data to a reference. We will demonstrate how Deming regression and bootstrapping techniques are used to validate NIRS based tissue oximeters, and the potential advantages. Bootstrapping validation allows pooling of all subject data to a best fit model used to set algorithm parameters and then performing model validation. Previous NIRS validations relied on methods involving splitting the subjects to two groups, calibration set and test set, and/or using Bland–Altman in various forms.

## Methods

### Technical and physiological background

The methodology of NIRS based tissue oximeters are well explained elsewhere [26–36]. In short, tissue oxygen saturation (StO_2_) measurements are based on the fact that oxyhemoglobin (HbO_2_) and deoxyhemoglobin (Hb) have different light absorption spectra. For brain, NIR light easily passes through skull bone and the absorption of NIR light by brain tissue is dependent upon chromophores (light absorbing tissue) within the path of the transmitted light in a highly optical scattering medium. HbO_2_ and Hb are the primary chromophores that absorb light, but background tissue light absorption can have a high impact on the measurement. Because biological tissue highly scatters light as well, reflectance type NIRS sensors can be used to target large organs such as the brain.

The first-generation FORE-SIGHT (CAS Medical Systems, Branford, CT USA) monitor was the second FDA cleared cerebral and somatic tissue oximeter to be widely available for clinical use in the USA, following the INVOS^®^ (Somanetics/Covidien, Boulder CO, USA) series of monitors. The first-generation FORE-SIGHT monitor used a laser light source with four discrete wavelengths (690, 780, 805, 850 nm) compared to INVOS using two LED light source wavelengths (730 and 810 nm). Besides accounting for HbO_2_ and Hb, the extra wavelengths used in FORE-SIGHT allowed for compensation for tissue background optical properties such as skin pigmentation and deep tissue optical characteristics, which can be highly variable among different human subjects. The next generation FORE-SIGHT ELITE^®^ tissue oximeter features a five wavelength LED light source (685, 730, 770, 805, & 870 nm). The purpose of the extra fifth wavelength was to further improve compensation for tissue background optical properties, as well as reduce measurement error due to the LED’s inherent wider spectral bandwidth. The algorithms used are based on a form of the Modified Beer–Lambert Law and are described elsewhere [7, 36, 37].

The monitor’s adult *Large* sensors have two detectors (near and far), where the far detector is 5.0 cm and near detector is 1.5 cm from the light source. The 5 cm far detector optode separation was selected as a tradeoff of having sufficient signal to noise ratio of detected light and sampling a higher percentage of brain tissue both in depth and in volume compared to smaller optode separations [38–43]. The 1.5 cm near detector optode separation was selected to sample extracerebral tissues, while minimizing brain tissue sampling [39]. The signals from the near detector are effectively subtracted from the far detector to minimize effects of extracerebral interference from blood and skin pigmentation, as well as to compensate for light source variability in the calculation of StO_2_ by a variant of the commonly used NIRS spatially resolved spectroscopy (SRS) method [44–48]. However, under extreme manipulations to separate brain and extracerebral compartments, full extracerebral interference elimination is not achieved [49–51]. Skin pigmentation and deep tissue optical characteristics still need to be further compensated by the aforementioned addition of extra wavelengths. Because human scalp and skull thicknesses can vary considerably [52–55], the 5 cm far detector optode spacing better accommodates anatomical variations with the increased interrogation depth over smaller optode separations [39, 40, 56].

Because NIRS technology mainly interrogates the microvasculature of tissue, which includes arterioles, venules, and capillaries, and does not involve the pulsatile signal component, a NIRS tissue oxygen saturation (StO_2_) measurement is made on a mixture of both venous and arterial blood. The general assumption used in our analysis is that mean ratio of this mixture for brain is estimated to be a ratio of 70% venous to 30% arterial blood by volume [11]. Whole body tissues are also estimated to contain the same mean ratio of 70% venous to 30% arterial blood by volume [24]. Therefore, to validate NIRS, oxygenation measurements of both venous and arterial blood need to be weighted from the venous output and arterial inputs of a target organ. For the brain, arterial blood supply is primarily from the carotid arteries and the primary venous drainage is by the internal jugular vein/jugular bulb. For somatic tissue, arterial blood supply is primarily from the descending aorta and the primary venous drainage is the vena cava leading to the right atrium. Because arterial blood oxygenation is similar in the larger blood vessels in the absence of congenital defects, blood was drawn from a catheter placed in the radial artery.

### Study protocol

The goal of this study was to evaluate the performance of the FORE-SIGHT ELITE in healthy volunteers during a controlled hypoxia sequence at steady-state ETCO_2_ levels to measure cerebral and somatic StO_2_ with the adult *Large* sensors. Subjects were healthy adult volunteers who were compensated for their study participation. Written informed consent was obtained from each subject prior to the initiation of any pre-study examination. Subjects were enrolled in either the cerebral or somatic cohort as venous catheter placement could only be in one location. For cerebral, a catheter was placed in the right jugular bulb for blood sampling, with location verified by X-ray. For somatic, a catheter was placed for blood sampling in the superior vena cava outside of the right atrium.

For cerebral StO_2_ validation, a *Large* sensor was placed on the left or right forehead close to the hairline, with placement alternated by even or odd subject number. The monitor’s values were compared to the calculated cerebral oxygen saturation (derived from co-oximeter measured arterial and jugular bulb venous oxygen saturations) during the sequential desaturation study.

For somatic StO_2_ validation, three *Large* sensors were placed on the flank, quadriceps, and calf muscles preferably at a high muscle density location. Sensor placements were alternated on the left or right side by even or odd subject number. The monitor’s values were compared to the calculated somatic oxygen saturation (derived from co-oximeter measured arterial and central venous oxygen saturations) during the sequential desaturation study.

The level of oxygen within the blood was reduced in a controlled manner by altering the inspired oxygen concentration (FiO_2_) to achieve arterial oxygen saturation plateaus between 100 and 70% as measured by a finger pulse oximeter on the finger. An attending anesthesiologist was present for each individual study. The anesthesiologist continuously monitored subject’s safety and managed the conduct of the study protocol. The subject’s tolerance of the study procedures was continually assessed and, if necessary, the study was prematurely terminated by subject request or clinical discretion.

**Fig. 1:**
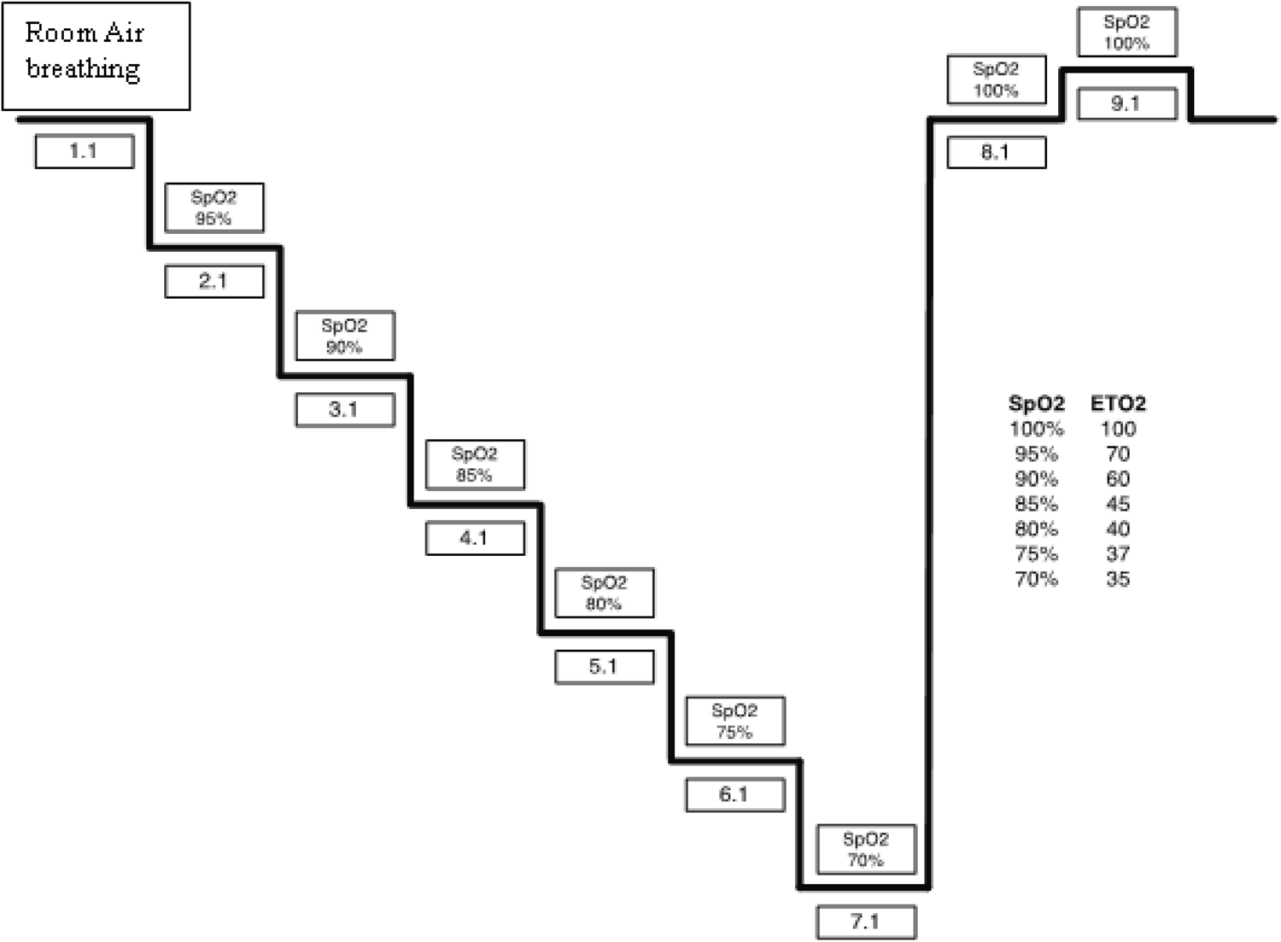
Stepped Hypoxia Plateau Sequence Protocol with targeted pulse oximetry SpO_2_ values and estimated ETO_2_ values previously derived experimentally from SpO_2_. For the NIRS cerebral StO_2_ validation portion of the protocol, jugular venous and arterial blood samples are drawn for co-oximetry analysis when the cerebral StO_2_ value stabilizes for each step. Likewise for NIRS somatic StO_2_ validation portion of the protocol, central venous and arterial blood samples are drawn for co-oximetry analysis when the somatic StO_2_ values from flank, quadriceps, and calf muscle stabilize for each step

First, the controlled hypoxia evaluation was conducted. The level of oxygen within the blood was reduced in a controlled manner by the RespirAct^®^ (Thornhill Research, Toronto, Canada) sequential gas delivery system (consisting of gas tanks, gas blender, facemask and control laptop with continuously displayed O_2_ and CO_2_) which permitted independent control of the both the ETO_2_ and ETCO_2_ to reach target values. ETCO_2_ was regulated to a target of 40 mmHg (±2) to normalize cerebral vasoreactivity to CO_2_ among subjects, to minimize changes in the venous and arterial blood volumes in cerebral tissue. The measured ETO_2_ and ETCO_2_ were used to closely match the target arterial O_2_ and CO_2_ (PaO_2_ and PaCO_2_), respectively. The PaO_2_ in turn determined the resultant SpO_2_. One (1) room air and eight (8) ETO_2_ plateaus were targeted, with resulting eight SpO_2_ plateaus between 70–100% (Fig. 1). At each plateau a set of paired arterial and jugular venous blood gas samples were drawn in heparinized syringes approximately 5 min after the FiO_2_ step, when the SpO_2_ and StO_2_ values stabilized. Blood samples were processed by a co-oximeter (ABL 90, Radiometer, Copenhagen, Denmark) to measure the arterial (S_a_O_2_) and jugular venous (S_jv_O_2_) oxygen saturations. Tissue oximetry measurements and blood samples were time-synchronized at each plateau. ECG, pulse, blood pressure, SpO_2_, ETCO_2_, and ETO_2_ were monitored throughout the study.

Institutional Review Boards (IRB) and Ethics Committees will only allow healthy volunteers to participate in low risk studies. The placement of the IJV bulb catheter represents a potential risk to healthy volunteers, though the precise risk of complications is difficult to quantify. Jugular bulb catheters have been used not only in healthy volunteers for oximeter validation studies but also for high altitude [57] and breath hold diving studies [58] without reported complications. In neuro-intensive care and neurosurgical patients several studies [59–63] have reported the safe use of jugular bulb catheters for clinical monitoring purposes. In the study by Coplin et al. [64], the authors reported a 40% incidence of thrombus at or near the site of line placement following line removal from 44 neuro-intensive care patients. Of particular note, the median duration of jugular line monitoring was 3 days and all the thrombi were considered subclinical with no patient experiencing symptoms. Therefore, we consider that the placement of IJV bulb catheters in healthy volunteers by experienced medical personnel using ultrasound guidance and for short periods of data collection (typically less than 4 h) to be appropriate and to constitute a low risk to study participants. There are published studies conducted under similar conditions to those defined for this study that demonstrate that healthy subjects tolerate the mild hypoxia and jugular bulb catheterization well without adverse outcomes [1, 2, 9, 10, 12].

Standard clinical procedure should be followed when performing jugular bulb catheterization to minimize risks [65, 66]. This study employs methods similar to the standard protocols recommended by the FDA and ISO Standards for testing pulse oximeters with mild hypoxia steps and blood drawn from arterial catheters (ISO 80601-2-61:2011) [67].

### Data analysis & statistical considerations

The monitor’s forehead cerebral StO_2_ measurements were compared to the weighted co-oximetry reference (REF CX_B_) based on the assumed 70:30 brain tissue venous:arterial (V:A) blood volume ratio [11] from the following equation [7]:

$${\text{REF C}}{{\text{X}}_{\text{B}}}={\text{ }}(0.7{\text{ }} \times {S_{jv}}{O_2}){\text{ }}+{\text{ }}(0.3{\text{ }} \times {S_a}{O_2})$$

where S_jv_O_2_ and S_a_O_2_ are the functional oxygen saturations from the blood samples drawn simultaneously from the internal jugular venous (jugular bulb) and systemic arterial catheters, respectively, and measured using a co-oximeter.

The monitor’s somatic StO_2_ readings were compared to the weighted co-oximetry reference (REF CX_S_) based on the assumed 70:30 somatic tissue V:A blood volume ratio [24] from the following equation:

$${\text{REF C}}{{\text{X}}_{\text{S}}}={\text{ }}(0.7{\text{ }} \times {S_{cv}}{O_2}){\text{ }}+{\text{ }}(0.3{\text{ }} \times {S_a}{O_2})$$

where S_cv_O_2_ and S_a_O_2_ are the functional oxygen saturations from the blood samples drawn simultaneously from the vena cava (near the right atrium), and systemic arterial catheters, respectively, and then measured using a co-oximeter.

We performed the Bland–Altman analysis [68] to evaluate the agreement between the measured StO_2_ and the REF CX, overall and within subgroups (e.g., light, moderate, and dark skin tones). Bias and precision (1 SD) were reported. Since the study design included repeated measurements within the same patient, a modified Bland–Altman analysis taking into account the repeated measures was performed using the MethComp package in R (http://BendixCarstensen.com/MethComp). We categorized Caucasian (White) subjects as having *light* skin tone, Asian and Hispanic subjects as having *moderate* skin tone, and African American (Black) subjects as having *dark* skin tone. For accuracy determinations, multiple subject data points were not binned like the alternative method of Ikeda, et al. [10]. We used both the random coefficients model and the Deming regression to estimate the intercept and slope (with 95% confidence intervals) of the measured StO_2_ against REF CX, following that of past FDA accepted 510 K clearances. The FDA favors the use of Deming regression [69], because it accounts for errors in observations on both the x-axis (REF CX reference) and the y- axis (measured StO_2_). The advanced regression methods add value by demonstrating the robustness of the validation. Results between the Deming regression and the traditional linear regression allow visual comparison to demonstrate consistency and similarity of the two methods to compensate for any possible weaknesses of each method. Also presented is the concordance correlation coefficient (CCC) [70], which is similar to the Pearson’s correlation coefficient but consists of a measure of precision multiplied by a measure of accuracy. Historically, successful cerebral oximeter FDA 510 K applications using a similar healthy adult hypoxia protocol have been based upon data from 17 to 23 subjects [71–73]. Power analysis shows that under the assumption that the true precision is 3%, there is an 80% chance that an experiment with 24 subjects will reliably detect an observed precision of 3.8% or less.

### Dependent data considerations

Because each subject had 9 data points each, the data are not independent. Therefore, a more complex analysis is detailed as follows. *Deming regression* assumes the reference (REF CX) is subject to measurement error. A Deming regression line will be fitted for each subject resulting in 25 regression lines with slope and intercept for the 25 subjects. Initial Q statistic analysis showed that the estimated standard errors for the regression coefficients are not homogeneous as some subjects have larger variation than others due to subject effect (not pure instrumental random error). This factor is taken into account when the estimates of intercepts and slopes are “pooled” together as part of a two-step process. Specifically, in Step 1, the Deming regression coefficients are determined for each subject, and then their standard errors are determined using the Jackknife method [74, 75]. In Step 2, the “Meta Analysis” technique is used to pool these estimates together to generate a weighted average intercept and a weighted average slope [76]. The pooled analysis considers that subjects are random samples from a general population.

*Random Coefficients Model* is a traditional method of linear regression and an alternate technique for analysis of subject dependent data. It assumes that each subject has his/her trajectory or inherent trend of the repeated measurements. The inherent trajectories are “high” or “low” with different steepness across subjects, suggesting that the subject-specific intercept and slope [77]. The concordance correlation coefficient (CCC) was used to demonstrate agreement for continuous data in this model. It can be used in the context of multiple repeated measurements per subject, and thus is valid for this study. [78].

### Bootstrap model validation

Bootstrapping is a statistical method for estimating the sampling distribution of an estimator by sampling with replacement from the original sample, with the purpose of deriving robust estimates of standard errors and confidence intervals of a population parameter such as regression correlation coefficient and confidence intervals. The regression methods (*Deming regression* and *Random Coefficients Model*) and CCC calculation were validated by two bootstrapping methods, *Bootstrap I* and *Bootstrap II*. The Bootstrap 95% confidence interval (CI) method was computed two different ways: “Normal”—normal approximation; and “Bias Corr”—bias-corrected percentile method. For *Bootstrap I*, individual subject data was bootstrapped without doing any moving block bootstrapping for repeated measurements within subjects. Sherman and le Cessie [79] present an ‘all block bootstrap’ by resampling blocks of individual subjects. They argued that, by bootstrapping these blocks, the correlation structure within each block could be maintained and the bootstrap intervals could be produced in an automatic way so that the correlation structure can be left unspecified. For *Bootstrap II*, we performed a second bootstrap method which does double bootstrapping for panel data (i.e., bootstrap for both subject and time series data) based on Davison and Hinkley [80]. For this method, we bootstrapped individual subjects first and then bootstrapped observations within individual subjects using the moving block method with block size 3 and overlap size 2. This is determined from the dataset, where there were 9 observations per subject where the desire block size is n^**(1/3)^ which is 9^**(1/3)^ ~ 2 or 3.

### Uncertainty analysis

Because it is likely that the assumed 70:30 brain tissue V:A volume ratio varies between subjects and with physiological conditions [81], weighted cerebral co-oximetry reference (REF CX_B_) based on the StO_2_ values were also compared to other brain tissue V:A volume ratios from 60:40 to 80:20 to quantify potential errors due to varying V:A volume ratios.

## Results

### Cerebral validation study

With informed consent, 25 subjects successfully completed the cerebral validation study with the following demographics: 15 White, 5 Black, 4 Asian, and 1 Hispanic subject, with 12 Males and 13 Females. Weight range was 44.6–108.9 kg; and age range was: 19.4–41.7 years.

Nine data points were obtained per subject from each FiO_2_ step along with REF CX_B_ for a total of 225 paired data points.

**Fig. 2:**
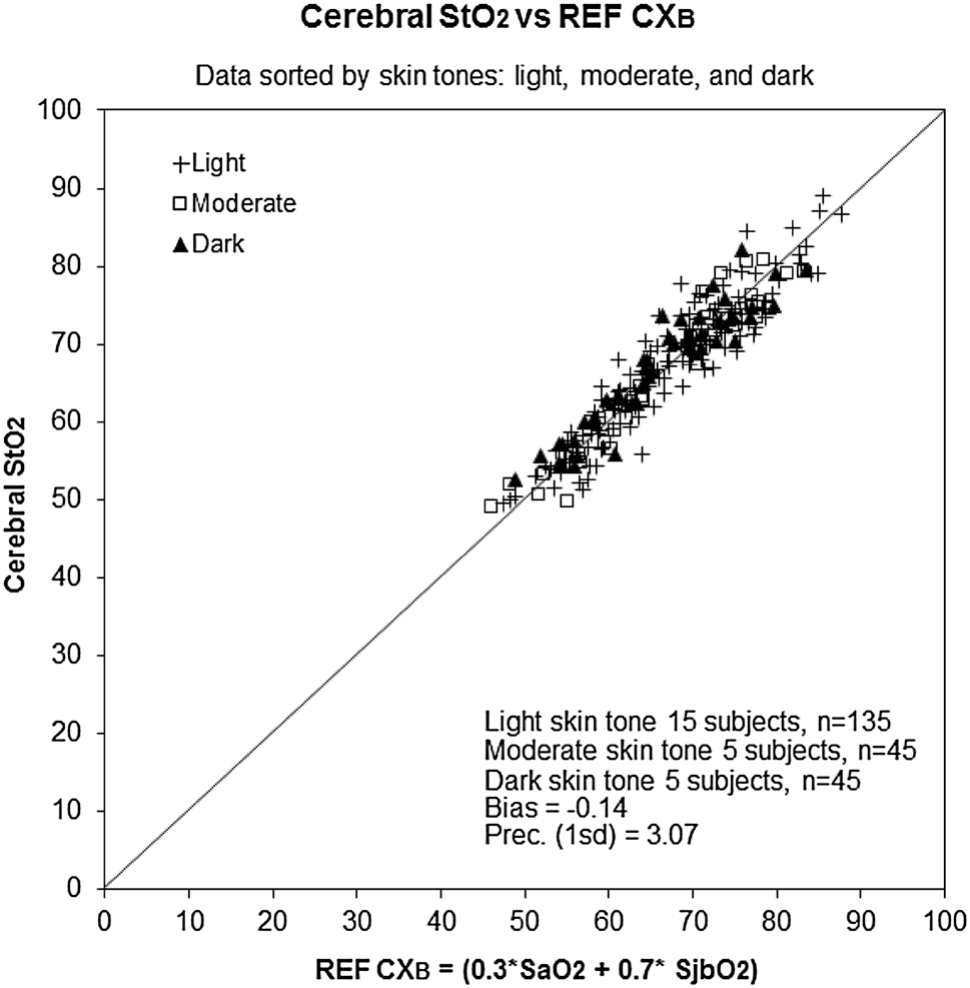
Scatter plot comparison of cerebral StO_2_ measurements to co-oximetry reference (REF CX_B_) with data points marked by skin tone (*dark*, moderate, and *light*)

The bias and precision (1 SD) of the monitor’s cerebral StO_2_ measurement vs REF CX_B_ for the three skin tone groups (light, moderate, and dark skin tones) are shown in Table 1. Figure 2 shows a scatterplot of the individual data points for the three skin tone types.

**Table 1:** Accuracy performance indicated by **(**Bias ± precision, 1 SD) of the monitor’s cerebral StO_2_ measurements versus REF CX_B_ for the three skin tone groups (light, moderate, and dark skin tones)

Skin tone	Subject (N)	Data points (N)	Deming regression equation	Cerebral StO_2_ (Bias ± 1SD)*
All	25	225	StO_2_ = 2.45 + 0.97 REF	−0.14 ± 3.07 (3.05)
Light	15	135	StO_2_ = 0.30 + 0.99 REF	−0.09 ± 3.27 (3.23)
Moderate	5	45	StO_2_ = 2.54 + 0.95 REF	0.52 ± 2.59 (2.53)
Dark	5	45	StO_2_ = 6.96 + 0.92 REF	−0.96 ± 2.90 (2.86)

Comparison of *Light* versus *Moderate* tone and *Light* versus *Dark* tone were not statistically significant (P > 0.05, t-test for means)

*Precision reported using Modified Bland–Altman for dependent data, with the more commonly reported standard Bland–Altman precision shown in parentheses

The monitor’s StO_2_ measurements from the right forehead sensor demonstrated an overall bias ± precision (1 SD) of 0.03 ± 3.02% (12 subjects), while the left forehead sensor demonstrated an overall bias ± precision (1 SD) of −0.30 ± 3.13% (13 subjects). Cerebral StO_2_ accuracy of both cerebral hemispheres was similar, even though jugular bulb catheterization was always on the right side.

The cerebral StO_2_ versus REF CX_B_ Deming regression was y = 0.966x + 2.447 and Random Coefficients model was y = 0.977x + 1.728 demonstrating the similarity of the results using subject dependent data regression techniques. The Concordance Correlation Coefficient (CCC) was 0.948. Using the rigorous Bootstrap techniques (Bootstrap I and Bootstrap II) for model validation, the Deming regression, Random Coefficients model, and CCC parameters were very similar to the observed values. The confidence intervals increase slightly when using the Bootstrap methods (Table 2).

**Table 2:** Comparison of cerebral StO_2_ measurements to co-oximetry reference (REF CX_B_): Deming and Random Coefficients Model Regression, CCC, and Bootstrap I and Bootstrap II Validation

Method	Original or bootstrap (bias–corrected) statistic	Standard error	Lower 95% confidence limit	Upper 95% confidence limit
Deming regression				
Intercept				
Observed	2.447	1.849	−1.176	6.071
Bootstrap I	2.511	2.626	−2.093	7.937
Bootstrap II	3.029	2.967	−2.921	8.368
Slope				
Observed	0.966	0.0287	0.909	1.022
Bootstrap I	0.964	0.040	0.882	1.041
Bootstrap II	0.958	0.046	0.878	1.054
Random coefficients model				
Intercept				
Observed	1.728	2.450	−3.328	6.784
Bootstrap I	1.800	2.464	−2.766	6.654
Bootstrap II	1.783	2.603	−3.773	6.763
Slope				
Observed	0.977	0.0390	0.897	1.058
Bootstrap I	0.976	0.039	0.900	1.055
Bootstrap II	0.976	0.041	0.898	1.062
Concordance correlation coefficient				
(CCC)				
Observed	0.948	0.015	0.877	0.960
Bootstrap I	0.954	0.023	0.891	0.975
Bootstrap II	0.916	0.008	0.929	0.929

Bootstrap I: Single Bootstrapping for dependent data—bootstrap individuals only and then sample entire block for each selected subject

Bootstrap II: Double Bootstrapping for dependent data—bootstrap individuals and then bootstrap observations within individuals using the moving block method with block size 3 and overlap size 2

Bootstrap CI method: bias-corrected percentile method

Bootstrap sample = 500

The cerebral StO_2_ values were compared to various weighted cerebral co-oximetry reference (REF CX_B_) in the uncertainty analysis. For brain tissue V:A volume ratios of 60:40, 65:35, 70:30, 75:25, and 80:20, the bias and precision (1 SD) of StO_2_ was 2.87 ± 2.82%, 1.37 ± 2.92%, −0.14 ± 3.05%, −1.65 ± 3.20%, and −3.16 ± 3.37%, respectively. The bias of StO_2_ versus REF CX_B_ changes about 0.30% per one point shift in the V:A volume ratio resulting in bias changes of ±3.0% for ratios of 60:40 or 80:20 the compared to the selected V:A ratio of 70:30. The mean SaO_2_–SjvO_2_ difference for all values was 30.15 ± 6.17 (1 SD).

### Somatic validation study

With informed consent, 24 subjects successfully completed the somatic validation study with the following demographics: 8 White, 14 Black, and 2 Asian, with 15 Male and 9 Female subjects. Weight range was 51.0–96.5 kg; and age range was: 19–40 years. Nine somatic StO_2_ measurements from the flank, quad, and calf (27 measurements total) were obtained per subject from the three sensors along with REF CX_S_.

**Fig. 3:**
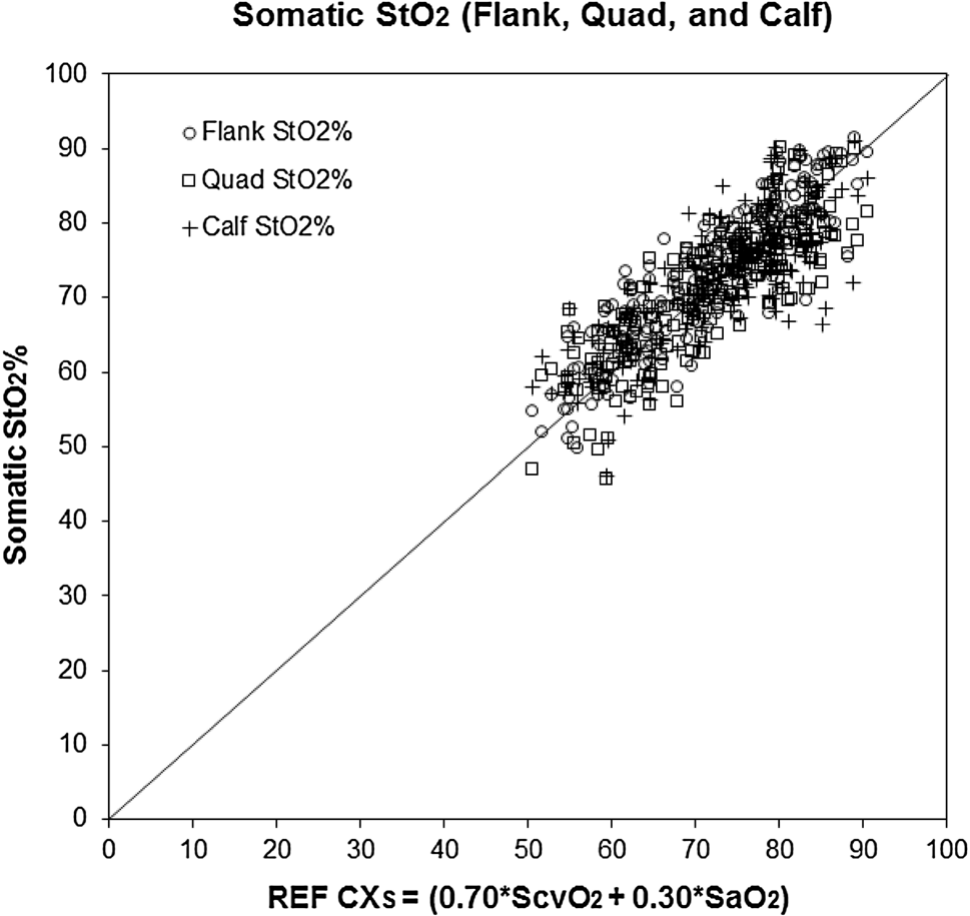
*Scatter plot* comparison of individual somatic StO_2_ measurements (*flank, quad*, and *calf*) versus Co-Oximetry Reference (REF CX_S_)

**Fig. 4:**
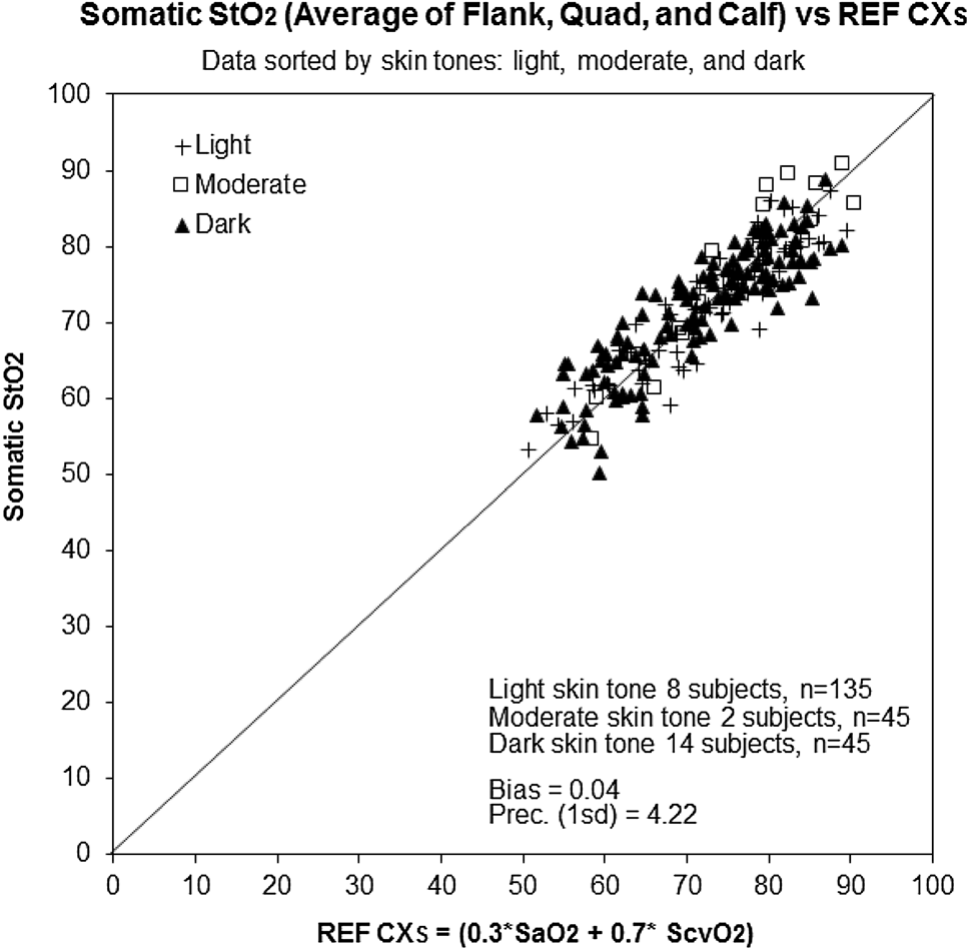
*Scatter plot* comparison of the average of *flank, quad*, and *calf* StO_2_ measurements to the global Co-Oximetry Reference (REF CX_S_), with data points marked by skin tone (*dark, moderate*, and *light*)

Figure 3 shows a scatterplot of StO_2_ versus REF CX_S_ for flank, quad, and calf overlaid. The bias and precision (1 SD) of separated flank, quad, and calf somatic StO_2_ versus REF CX_S_ are shown in Table 3. Figure 4 shows a scatterplot of StO_2_ versus REF CX_S_ for averaged flank, quad, and calf StO_2_ values at each blood draw time for the three skin tone groups (light, moderate, and dark skin tones). The bias and precision (1 SD) of StO_2_ average of flank, quad, and calf values for the three skin tone groups versus REF CX_S_ are shown in Table 3.

**Table 3:** Accuracy performance indicated by (Bias ± precision, 1 SD) of Somatic StO_2_ versus REF CX_S_ for the individual three body locations monitored (Flank, Quad, and Calf)

Skin tone	Body location	Subject (N)	Data points (N)	Deming regression equation	Somatic StO_2_ (Bias ± 1 SD)*
All	Flank	24	216	StO_2_ = 4.78 + 0.95 REF	−1.12 ± 4.45 (4.42)
	Quadriceps	24	216	StO_2_ = 7.54 + 0.88 REF	1.11 ± 5.41 (5.37)
	Calf	23	201	StO_2_ = 15.13 + 0.79 REF	0.03 ± 5.91 (5.88)
	Average of Flank/Quad/Calf	24	216	StO_2_ = 9.51+0.87 REF	0.04 ± 4.22 (4.20)
Light	Flank	8	72	StO_2_ = 1.17 + 1.00 REF	−0.66 ± 4.41
	Quadriceps	8	72	StO_2_ = 6.18 + 0.90 REF	1.58 ± 5.19
	Calf	8	72	StO_2_ = 20.84 + 0.71 REF	0.90 ± 4.63
	Average of Flank/Quad/Calf	8	72	StO_2_ = 9.26 + 0.87 REF	0.62 ± 3.77
Moderate	Flank	2	18	StO_2_ = 11.77 + 1.19 REF	−2.06 ± 3.61
	Quadriceps	2	18	StO_2_ = −9.39 + 1.11 REF	1.11 ± 5.12
	Calf	2	18	StO_2_ = −3.10 + 1.07 REF	−2.32 ± 4.31
	Average of Flank/Quad/Calf	2	18	StO_2_ = −7.21 + 1.11 REF	−1.09 ± 3.95
Dark	Flank	14	126	StO_2_ = 8.96 + 0.89 REF	−1.24 ± 4.64
	Quadriceps	14	126	StO_2_ = 10.43 + 0.84 REF	−0.84 ± 5.69
	Calf	13	117	StO_2_ = 16.25 + 0.77 REF	0.14 ± 6.74
	Average of Flank/Quad/Calf	14	126	StO_2_ = 11.59 + 0.84 REF	−0.13 ± 4.54

*Precision reported using Modified Bland–Altman for dependent data, with the more commonly reported standard Bland–Altman precision shown in parentheses for the All Skin Tone group

The averaged (flank, quad, & calf) somatic StO_2_ versus REF CX_S_ Deming regression was y = 0.867x + 9.514 and Random Coefficients model was y = 0.846x + 10.929 demonstrating the similarity of the results using subject dependent data regression techniques. The differences between the flank, quad, and calf in terms of individual Deming regression equations (Table 3) where quad and calf slopes are lower than flank, as well as bias & precision may have an influence on the overall regression slope and intercept when the data points are averaged. The Concordance Correlation Coefficient (CCC) was 0.821. Using the rigorous Bootstrap techniques (Bootstrap I and Bootstrap II) for model validation, the Deming regression, Random Coefficients model, and CCC parameters were very similar to the observed values. The confidence intervals increase slightly when using the Bootstrap methods. The averaged somatic site StO_2_ compared to the global REF CX_S_ accuracy was better than the individual somatic site StO_2_ measurements, and accuracy decreased as the somatic measurement body location was made farther away from the central venous REF CX_S_ blood draw location (Table 4). Also cerebral StO_2_ accuracy vs REF CX_B_ was better than somatic StO_2_ vs REF CX_S_.

**Table 4:** Comparison of average of flank, quad, and calf StO_2_ values to the global co-oximetry Reference (REF CX_S_): Deming and random coefficients model regression, CCC, and Bootstrap I and Bootstrap II Validation

Method	Original or bootstrap (bias–corrected) statistic	Standard error	Lower 95% confidence limit	Upper 95% confidence limit
Deming regression				
Intercept				
Observed	9.514	3.209	3.224	15.803
Bootstrap I	9.453	3.455	2.388	15.722
Bootstrap II	9.865	4.255	1.387	18.243
Slope				
Observed	0.867	0.043	0.783	0.952
Bootstrap I	0.868	0.046	0.784	0.958
Bootstrap II	0.863	0.057	0.748	0.976
Random coefficients model				
Intercept				
Observed	10.929	3.405	3.885	17.974
Bootstrap I	10.929	3.280	4.428	17.522
Bootstrap II	10.724	3.835	3.542	18.310
Slope				
Observed	0.846	0.046	0.751	0.940
Bootstrap I	0.845	0.044	0.760	0.936
Bootstrap II	0.847	0.052	0.746	0.949
Concordance Correlation Coefficient				
(CCC)				
Observed	0.821	0.047	0.706	0.894
Bootstrap I	0.840	0.067	0.677	0.917
Bootstrap II	0.680	0.014	0.880	0.880

Bootstrap I: Single Bootstrapping for dependent data—bootstrap individuals only and then sample entire block for each selected subject

Bootstrap II: Double Bootstrapping for dependent data—bootstrap individuals and then bootstrap observations within individuals using the moving block method with block size 3 and overlap size 2

Bootstrap CI method: bias-corrected percentile method

Bootstrap sample = 500

## Discussion

The validation methodology of tissue oximeters to invasive blood reference values assumes a fixed venous to arterial (V:A) blood volume ratio that can be applied to all subjects. The V:A blood volume ratio likely varies, with different analyses suggesting cerebral V:A blood volume ratios ranging from 54:46 to 84:16 [7, 82]. Because 70:30 is near the midpoint of the estimated V:A range [7] and imaging techniques also suggest the mean cerebral V:A blood volume ratio is approximately 70:30 among different subjects in steady state healthy conditions [11], we believe that an V:A ratio of 70:30 is a reasonable assumption for the brain. Our data indicates that if the actual V:A ratio varied 60:40–80:20 between subjects, the bias of StO_2_ versus REF CX_B_ would change ±3.0% compared to the selected V:A ratio of 70:30. The high precision of the FORE-SIGHT ELITE (3.07% 1 SD) for cerebral StO_2_ against the fixed 70:30 reference weighting across the StO_2_ 50–90% saturation range therefore suggests that for healthy subjects under controlled PaCO_2_ conditions, the inter- and intra-subject subject variability of V:A ratio is likely less than ±10%. As an indirect comparison, pulse oximetry precision for adults derived from a controlled hypoxia study is ~2% (1 SD) when compared to arterial blood oxygen saturation [83]. It is unlikely that in-vivo validated NIRS tissue oximetry systems will reach pulse oximeter precision, in part because NIRS tissue oximeters need both arterial and venous blood oxygen saturation co-oximeter measurements, which adds more variability to the REF CX reference measurement, and also because NIRS tissue oximetry interrogates deeper into tissues to make a StO_2_ measurement. Note that an NIRS monitor cannot measure the actual V:A blood volume ratio in tissue and does not distinguish venous and arterial contributions, a common point of confusion of NIRS monitors. The V:A ratio is only used to derive a reference from blood samples during validation of the NIRS monitor.

An interpretation of this data is that the inter-subject variability of cerebral vasoreactivity during controlled PaCO_2_ conditions is likely low within healthy adult subjects. The mean V:A ratio will then be likely less variable compared to other patient populations with morbidities or during uncontrolled PaCO_2_ states. Therefore, validation with healthy adult subjects with controlled PaCO_2_ may serve as a control. Measured precision and regression parameters would then be indicators on how the tissue oximeter performs under near-ideal conditions. A tissue oximeter that shows more variability when compared to a reference under near-ideal conditions, will likely demonstrate more variability when used as a clinical monitor. A controlled tissue oximetry validation cannot be performed for pediatric and neonatal subjects for ethical reasons and so non-healthy pediatric subjects undergoing cath-lab procedures are commonly used [13, 23, 84, 85]. As a result, precision and regression parameters from pediatric tissue oximetry validation exhibit more variability compared to a control study [13, 85]. Because tissue oximetry general sensor and algorithm designs are usually similar for a particular model tissue oximeter among different subject populations, the adult validation may indirectly serve as a reference for pediatric tissue oximetry performance as well.

It is understood that the cerebral venous to arterial blood volume ratio varies physiologically in the tissue vasculature that is interrogated by a NIRS sensor [7, 86–88] as PaCO_2_ normally varies among human and other mammalian subjects. Since CO_2_ is a potent vasodilator to the cerebral vasculature, PaCO_2_ levels in blood can shift the V:A ratio where high PaCO_2_ levels (hypercapnia) would drive arterial blood volume ratio to be greater than 30% while low PaCO_2_ levels (hypocapnia) would drive arterial blood volume ratio to be less than 30% [89, 90]. Because hypocapnia results in vasoconstriction of cerebral arterial blood vessels, resulting in reduced flow, cerebral tissue ischemia can result [91–95]. In addition to the effects of lower perfusion, a NIRS sensor would also interrogate less arterial blood volume relative to venous blood volume in the tissue. This compound effect will result in a decrease of StO_2_, which would alert the clinician and warrant a check in PaCO_2_ levels [96–98]. Reduced minute ventilation to increase CO_2_ levels is often used as an intervention to increase cerebral blood flow and resultant perfusion [99–103]. In this case, a NIRS sensor would detect an increase of arterial blood volume relative to venous blood volume as well as an increase in flow resulting in an increase of StO_2_, the desired effect. Therefore, we believe that a cerebral tissue oximeter validated using a controlled fixed V:A blood volume ratio REF CX_B_ reliably provides clinicians real time information of the effect of both adverse and beneficial changes in cerebral vasoreactivity and V:A blood volume ratio shifts.

For the somatic co-oximeter reference REF CX_S_, the mean V:A non-cerebral tissue blood volume ratio was also assumed to be 70:30 among different subjects in steady state healthy conditions. This assumption was based on the findings of Pang et al. [24] where the venous system of the whole body contains 70% of total blood volume. However, somatic tissue blood volume V:A ratios can vary greatly under normal and abnormal physiological conditions. For example, muscle exercise may dynamically change V:A ratio between contraction and relaxation. Body position, such as standing upright, may result in pooling venous blood volume in the lower extremities compared to the supine position. Therefore, for somatic validation, the subjects were in the supine position and relaxed, with negligible muscle activation resulting in resting state metabolism for the somatic sensor measurement sites. This controlled resting state appeared to effectively limit the variation in V:A blood volume ratio as evidenced by somatic StO_2_ accuracy measurements within 6% (1 SD) compared to a fixed 70:30 blood volume ratio REF CX_S_.

The results showed that the somatic StO_2_ measurement precision and individual Deming regression slope decreased as the body location moved farther away from the heart compared to REF CX_S_. The Flank StO_2_ measurements showed the highest precision (4.45%), followed by Quad StO_2_ measurements (5.41%), then Calf StO_2_ measurements (5.91%). Because the blood in the vena cava represent the global venous blood return of the body, multiple somatic StO_2_ measurements are averaged to better reflect the global SvcO_2_ co-oximetry measurement as part of REF CX_S_, with a precision of 4.22% compared to the next best 4.45% of the Flank StO_2_ measurements alone. Due to heterogeneity in tissue oxygenation demand and metabolism, it is likely that somatic StO_2_ would have some variability at different body locations. An alternative validation method for limb muscle StO_2_ is to use blood from the venous return of the limb that is close to the muscle of interest [104] as opposed to the global vena cava venous return done in this study. Somatic StO_2_ measurements are best made on the larger muscles of the body, where NIRS light can diffuse and scatter unimpeded by the tissue geometry. Bony areas of the body such as ankles, wrists, and parts of the hands and feet, may alter the NIRS photon path to the sensor detectors, resulting in unreliable StO_2_ measurements, particularly with larger light source to detector configured sensors.

When validating tissue oximetry data to an internal blood reference, two different data analysis methods accepted by the U.S. FDA can be chosen. The first method involves splitting the subjects to two groups, calibration set and test set [12]. The second method involves pooling all subjects to a best fit model used to set algorithm parameters and then doing model validation using statistical techniques such as bootstrapping, which was done here. To determine which validation method to use, the following considerations need to be examined. For clinical validity and generalizability, the enrolled subject group should reflect those of the general population in terms of demographics such as weight, gender, and skin pigmentation. In a recent FDA guidance for pulse oximetry, the FDA recommends use of a minimum of 200 paired data points from at least 10 subjects where at least 2 subjects or 15% of subjects are darkly pigmented, whichever is larger [83]. Besides skin pigmentation, inter-subject variability of deeper tissue background optical properties can have an impact on tissue oximeter accuracy when compared to a blood reference. Such inter-subject differences have been observed to result in physiologically anomalous readings or variable agreement to invasive blood references [12]. Deep tissue optical characteristics may include the optical effects of tissue, muscle, and bone density, heterogeneous tissue pigmentation, hair follicles, and scarring from prior injuries, contusions, concussions, or facial surgeries. Furthermore, anatomical variations influence the distribution and characteristics of the various tissue contributions. Since the background deep tissue optical characteristics cannot be determined by visually examining subjects and are independent of race, an effective sample size needs to have a high probability to include a wide range of subjects with different deep tissue optical characteristics.

Two follow-up first generation FORE-SIGHT studies with comparison to the invasive reference REF CX_B_ [105, 106] showed consistency in precision following validation using the modeling and statistical validation method with 17 subjects [71]. The validation of another tissue oximeter using the calibration and test method splitting 23 subjects in two groups (11 calibration subjects and 12 test subjects) [12] gave an unexpected result where the test accuracy measurement was better than the calibration value, which may indicate that the test group subjects had less background tissue optical heterogeneity than the calibration group. For this reason, the approach described herein using the full data set for the best fit modeling and advanced statistical validation techniques was chosen for the FORE-SIGHT ELITE. By using a larger data set and accounting for sampling variability, this method may be more reliable in predicting clinical monitor performance over a wider range of subjects with different background optical characteristics. For validations done using the split subject datasets to two groups (calibration set and test set) to match the effective sample size that includes a wide range of subjects with different deep tissue optical characteristics, the overall effective subject sample size would need to be doubled.

When considering accuracy of NIRS tissue oximeters to other oximetry systems, the semi-invasive optical based SvO_2_ catheters may be the best for comparison. These catheters measure SvO_2_ in venous blood vessels around the heart (central venous) and internal jugular vein/jugular bulb, part of the brain venous drainage system. SvO_2_ catheters measure SvO_2_ directly with an optical interface to blood where light does not pass through tissues first like tissue oximetry. For three SvO_2_ catheter oximeter systems, in-vivo comparison with co-oximetry of blood samples demonstrated a precision of 4.3–7.1% (1 SD) [107]. For the Edward Lifesciences (Irvine, CA) Vigileo™ SvO_2_ catheter system, the in-vivo comparison with co-oximetry of blood samples demonstrated a precision of 4.1% (1 SD) [108, 109]. The precision of FORE-SIGHT ELITE StO_2_ for cerebral (3.07% 1 SD) and somatic (4.22% 1 SD) are very comparable to optical SvO_2_ catheter oximetry systems.

An alternative method in validating NIRS tissue oximeters under development involves in-vitro tests on a liquid optical phantom [110–114]. The liquid phantom contains a predetermined solution of saline, human blood hemoglobin, Intralipid^®^, sodium bicarbonate, glucose, and baker’s yeast to desaturate the hemoglobin [110, 111]. An issue that needs to be resolved is that different NIRS devices measure different StO_2_ values from sensors placed on the phantom and in-vivo validated NIRS monitors produce different values than those independently measured on the blood inside the phantom [110, 111]. This is in part due to the different algorithms of the monitors, the sensor optical configuration, how the monitors compensate for skin pigmentation and background optical properties other than hemoglobin, and the validation methodology of the monitor. Phantoms generally absorb and scatter light differently compared to that of tissue oximeter sensors placed on human subjects as evidenced by the attenuation of light from each sensor’s light source wavelengths (personal observation). If the optical properties of phantoms and biological tissue are not well matched, a tissue oximeter StO_2_ algorithm may behave differently, where the value and rate of change of StO_2_ compared to a phantom blood saturation reference will have a bias and different regression slope. One improvement in phantom design may include better optical spectral matching with human tissues for light attenuating components other than hemoglobin. Skin pigmentation and deeper tissue optical characteristics, which attenuate light more in the lower wavelengths <750 nm [115] could be added to the phantoms, perhaps as a red dye, to better model these tissue optical characteristics. An ideal phantom would give the same quantitative value for the tissue oximeter parameter of interest (such as StO_2_) when measured by different manufacturer model monitors, corresponding to the same quantitative parameter value measured on human subjects. In the future, an in-vivo blood co-oximetry validated monitor “A” could be used to calibrate the ideal NIRS phantom, then this phantom can be used to calibrate and/or test monitors “B”, “C” etc.

Tissue oximeter validation should be standardized so that in clinical use, StO_2_ measurements between tissue oximetry models are more consistent. Areas of standardization may include using a fixed mean blood volume ratio based on best available information for which we suggest using a blood volume V:A ratio of 70:30, use of highly accurate co-oximeter models especially at lower oxygen saturation values for the reference measurements, and for adult subjects, use of a hypoxia protocol with good distribution of FiO_2_ levels while controlling PaCO_2_ levels to a limited range. A good distribution of skin tones from the different races are needed [83] as well as obtaining randomly a good distribution of subject background optical characteristics by having an effective sample size. If a liquid or other optical phantom can model all these parameters, then an alternative NIRS validation method may be available in the future.

For direct comparisons of NIRS tissue oximeter models, caution is advised in interpreting the results when no comparative co-oximetry blood oxygen saturation reference (such as REF CX) is used as a control. One cannot determine which monitor is more accurate or has the more appropriate StO_2_ value or rate of change [116] during an hypoxic or ischemia event without an appropriate comparative reference. Likewise, caution is advised in interpreting comparisons of different NIRS tissue oximeter models to blood oxygen saturation references different from that of the original NIRS tissue oximeter’s validation reference such as cerebral StO2 vs central or mixed venous SvO_2_ [117–119]. Furthermore, results may not be comparable when the inappropriate sensor is applied outside the manufacturer’s indications for use such as an adult validated sensor to an infant subject [120]. Both the StO_2_ value and the rate of change of StO_2_ to a physiological event will likely be inaccurate as the assumptions behind the sensor design and algorithm used will be different.

Ultimately, demonstrated clinical utility of NIRS tissue oximeters is important to gain acceptance for use in patient monitoring in healthcare systems. Relationships between StO_2_ and both physiological parameters and outcomes variables have been discussed elsewhere [121, 122]. Low StO_2_ values has been associated with post-op complications in aortic surgery [123], single lung ventilation [124, 125], beach chair shoulder procedures [126], and in cardiac surgery [100, 101, 127]. StO_2_ values provide guidance of setting ventilation controls particularly end tidal CO_2_ [103], setting safe ablation and entrainment mapping periods in ventricular tachycardia treatment [128], targeting oxygen saturation ranges to reduce risk of retinopathy in neonates [129], and catastrophic avoidance such as detection of misplaced cannulas and incorrect ventilation settings in surgery [130–132]. More interventional studies are needed to see if goal directed therapy based on StO_2_ can improve outcomes [121]. Standardized validation of tissue oximeters allows for better cross analysis of data between different manufacturer monitor models increase the potential of finding clinical correlations with disease states, corresponding outcomes, and determining possible interventions to improve outcomes.

In conclusion, we present the validation of the FORE-SIGHT ELITE tissue oximeter and the rationale behind the assumptions made in the protocol based on our experience with these monitors. We assumed that the cerebral and somatic invasive blood reference consisting of weighted tissue mean blood volume ratio (V:A) is 70:30 at PaCO_2_ of 37–40 mmHg based on prior publications, and that this ratio is generally constant for healthy human subjects because of the high level of precision of tissue oximeter StO_2_ when compared to this invasive reference. We acknowledge that the V:A blood volume ratio normally varies in physiology and believe that monitoring StO_2_ is clinically important in part to show how the V:A ratio changes due to CO_2_ or other agents affecting tissue oxygenation. We believe that use of advanced statistical techniques such as Deming regression and bootstrap resampling to validate the best fit full data set model provides a more reliable representation of clinical performance over a wider range of subjects with different skin tones and background optical characteristics for a given sample size. Finally, we suggest standardization of tissue oximetry validation, whether in-vivo as presented, and/or in-vitro with an ideal NIRS phantom when perfected, so that tissue oximeters used in the clinic make more reliable measurements, with more consistency between different manufacturer tissue oximetry models, and therefore maximize overall utility of tissue oximetry in the clinic.
